# Defective neutrophil development and specific granule deficiency caused by a homozygous splice-site mutation in *SMARCD2*

**DOI:** 10.1016/j.jaci.2020.11.025

**Published:** 2021-06

**Authors:** Ina Schim van der Loeff, Evelien G.G. Sprenkeler, Anton T.J. Tool, Mario Abinun, Angela Grainger, Karin R. Engelhardt, Michel van Houdt, Hans Janssen, Taco W. Kuijpers, Sophie Hambleton

**Affiliations:** a Immunity & Inflammation Theme, Translational and Clinical Research Institute, Faculty of Medical Sciences, Newcastle University, Newcastle upon Tyne, United Kingdom; b Great North Children’s Hospital (GNCH), Newcastle upon Tyne Hospitals NHS Foundation Trust, Newcastle upon Tyne, United Kingdom; c Department of Blood Cell Research, Sanquin Research, Amsterdam University Medical Centre, University of Amsterdam, Amsterdam, The Netherlands; d Department of Pediatric Immunology, Rheumatology and Infectious Disease, Emma Children’s Hospital, Amsterdam University Medical Centre, University of Amsterdam, Amsterdam, The Netherlands; e Division of Biochemistry, The Netherlands Cancer Institute, Amsterdam, The Netherlands

**Keywords:** Splice-site mutation, CEBPε, lactoferrin, chemotaxis, neutrophil-specific granule deficiency, phagocyte disorder, inborn error of immunity, CEBPε, CCAAT-enhancer-binding protein ε, SGD, Specific granule deficiency, SMARCD2, SWI/SNF-related, matrix-associated, actin-dependent regulator of chromatin, subfamily D, member 2

## Abstract

**Background:**

SMARCD2 (SWI/SNF-related, matrix-associated, actin-dependent regulator of chromatin, subfamily D, member 2) has recently been shown to have a critical role in granulopoiesis in humans, mice, and zebrafish. Our patient presented with delayed cord separation, failure to thrive, and sepsis. Retrospective whole-exome sequencing confirmed a homozygous splice-site mutation in *SMARCD2*.

**Objective:**

We sought to provide the second description of human SMARCD2 deficiency and the first functional analysis of human primary SMARCD2-deficient cells.

**Methods:**

Heparinized venous blood and bone marrow were collected from the patient after obtaining informed consent. Patient leukocytes and CD34^+^ cells were then isolated, phenotyped, and assessed functionally.

**Results:**

Circulating neutrophils appeared phenotypically immature, lacking multilobed nuclei, and neutrophil granules lacked lactoferrin but showed normal levels of myeloperoxidase. Neutrophil oxidative burst was preserved in response to phorbol 12-myristate 13-acetate. Patient bone marrow–derived neutrophils and white blood cells showed a severely impaired chemotactic response. Furthermore, white blood cells showed defective *in vitro* killing of *Staphylococcus aureus*, consistent with a specific granule deficiency. Finally, patient bone marrow–derived CD34^+^ cells showed markedly impaired *in vitro* expansion and differentiation toward the neutrophil lineage. Before her molecular diagnosis, our patient underwent hematopoietic stem cell transplantation and is well 8 years later.

**Conclusions:**

This report highlights an important role for SMARCD2 in human myelopoiesis and the curative effect of hematopoietic stem cell transplantation for the hematopoietic features of SMARCD2 deficiency.

## Introduction

The chromatin-remodeling factor SMARCD2 (SWI/SNF-related, matrix-associated, actin-dependent regulator of chromatin, subfamily D, member 2) plays an important role in myeloid differentiation in humans. Similar to patients with specific granule deficiency (SGD),^1^^,^^2^ patients who lack SMARCD2 present with delayed cord separation, recurrent bacterial infections, and absent neutrophil granule proteins.^3^ In addition to regulating chromatin accessibility,^3^ SMARCD2 interacts with the transcription factor CCAAT-enhancer-binding protein ε^3^ and is essential for its recruitment to the promoter of neutrophilic-specific granule genes.^4^ Interestingly, mutations in CCAAT-enhancer-binding protein ε that result in SGD^1^^,^^2^ abrogate this interaction with SMARCD2, suggesting that at least some of the effects of SMARCD2 deficiency are mediated by CCAAT-enhancer-binding protein ε. Here, we present the first analysis of human SMARCD2-deficient primary cells and the second report of human SMARCD2 deficiency leading to neutrophil-specific granule deficiency.

## Results and discussion

**Fig 1:**
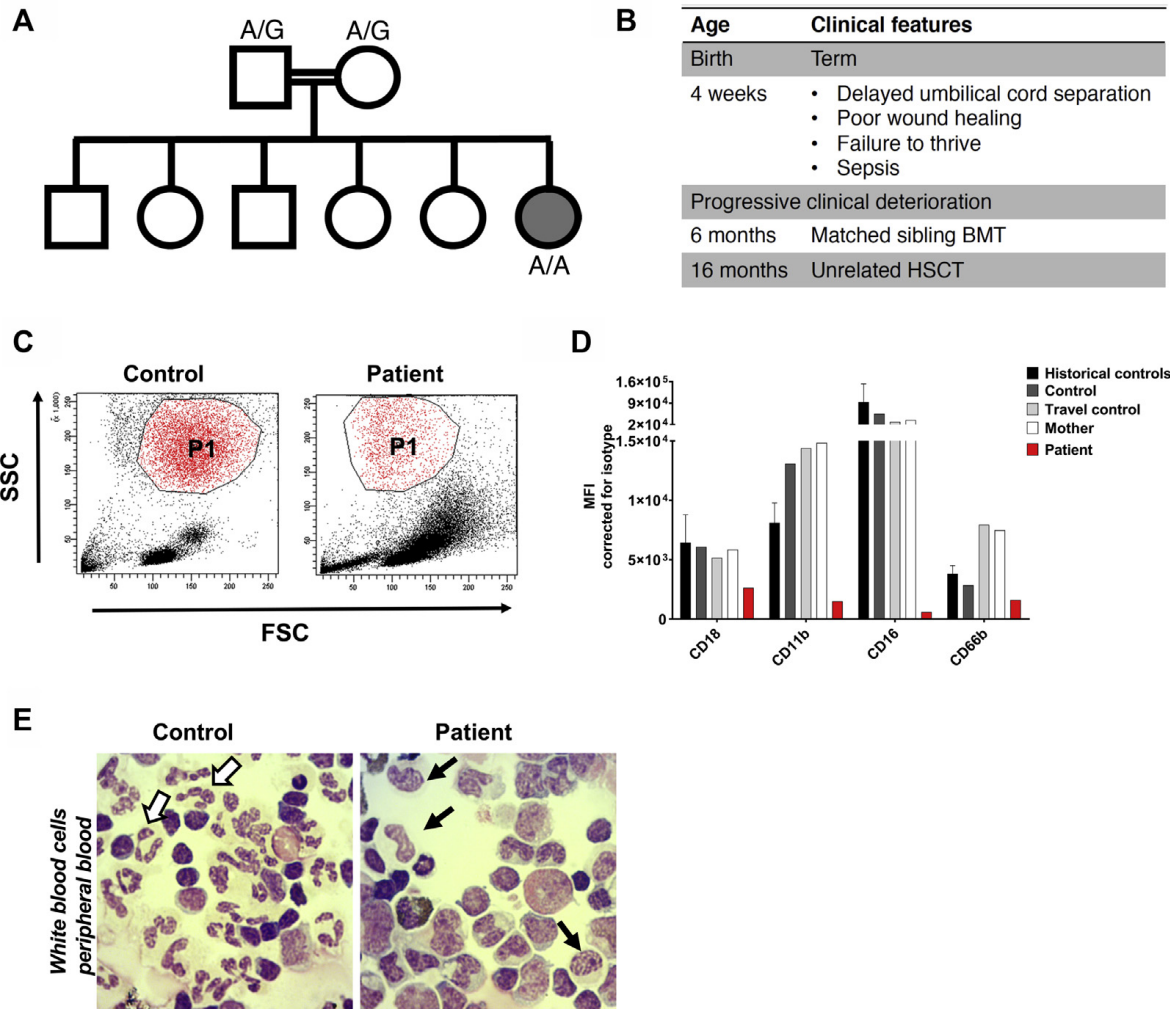
Primary SMARCD2-deficient neutrophils are phenotypically immature. **A,** Patient family tree. Double lines indicate consanguinity; circle indicates female; square indicates male; gray-filled circle indicates patient. The *SMARCD2* genotype at c.1181+1 is indicated for the proband and her parents. **B,** Patient clinical features. **C,** Flow cytometric gating strategy for neutrophils (P1) based on forward/side scatter (FSC vs SSC). **D,** Expression of neutrophil maturation markers on the cell membrane was assessed by flow cytometry. Neutrophils are gated as in Fig 1, *C* (n = 1). Historical healthy controls (1-day old blood) are also depicted (mean + SD, n = 9-13). **E,** Cytospins of isolated white blood cells. White arrows indicate normal segmented neutrophils (in the travel control, left), and black arrows indicate cells that morphologically resemble metamyelocyte- or band neutrophils (in the patient, right) (original magnification ×400; May-Grünwald Giemsa stain). *BMT*, Bone marrow transplant; *FSC*, forward scatter; *HSCT*, hematopoietic stem cell transplantation; *MFI*, mean fluorescence intensity; *SSC*, side scatter.

We investigated a patient who was transferred to our care at age 1 month with poor feeding, weight loss, a sublingual ulcer, and delayed cord separation with omphalitis. She had been born uneventfully at term by elective cesarean section, requiring no special care. She was the sixth child of consanguineous parents, and her 5 older siblings were well (Fig 1, *A*). In view of fever, she received broad-spectrum antibiotics for presumed infection. Cultures of blood and cerebrospinal fluid were sterile, but an umbilical swab yielded *Staphylococcus aureus* and omphalitis was confirmed histologically on the separated cord.

An inborn error of immunity such as leukocyte adhesion deficiency was suspected, and care was shifted to a specialist setting (Fig 1, *B*). Initial diagnostic workup revealed normal neutrophil counts and excluded classical type 1 leukocyte adhesion deficiency on the basis of weakly preserved expression of adhesion molecules CD11a, b and c, and CD18; a neutrophil oxidative burst was also demonstrated. However, neutrophil morphology was reported as abnormal, and these cells were difficult to gate (Fig 1, *C*). A bone marrow aspirate showed few mature neutrophils and abnormal granulation in the myeloid series. Other immunologic investigations including immunoglobulins and lymphocyte subsets were normal.

Over the following weeks, the patient remained unwell despite full supportive care. She had ongoing diarrhea and poor weight gain, in the absence of enteric pathogens and with minimal chronic inflammatory infiltrate on endoscopic gastrointestinal biopsies. She also suffered recurrent episodes of respiratory distress requiring supplemental oxygen, associated with bilateral consolidation and segmental collapse on high-resolution computed tomography. Bronchoalveolar lavage was negative for fungi, viruses, and bacteria, including opportunistic pathogens, as was a lung biopsy taken a few weeks later. However, the latter showed a mild to moderate cellular bronchiolopneumonitis with associated well-formed granulomas, presumably postinfectious or autoinflammatory in origin. The patient also manifested developmental delay disproportionate to her overall clinical picture with head lag, central hypotonia, and peripheral hypertonia at age 4 months. Cranial magnetic resonance imaging and electroencephalography were normal.

**Fig 2:**
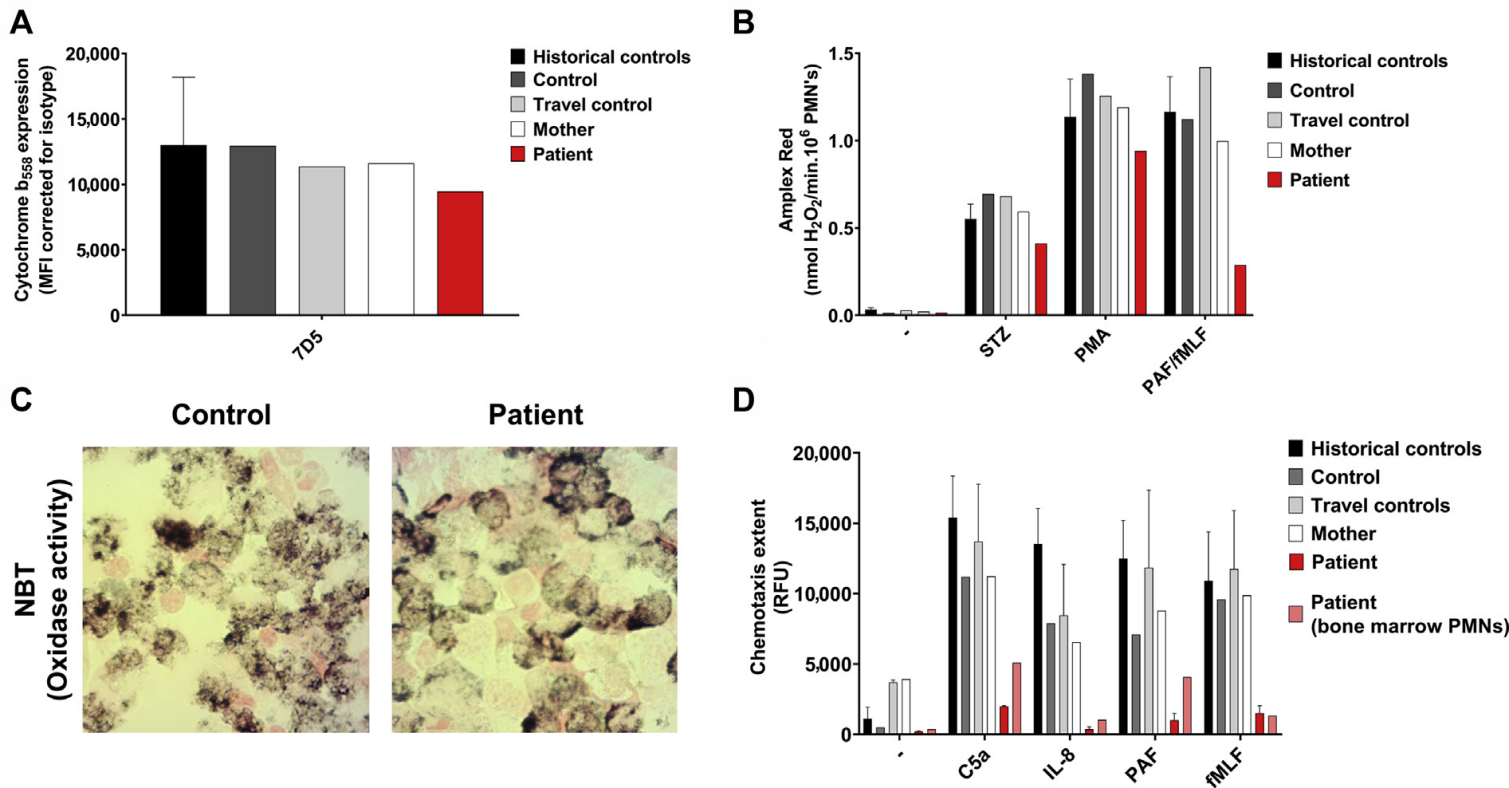
Neutrophil oxidative burst and chemotaxis are impaired in patient leukocytes. **A,** Expression of cytochrome b_558_ (the gp91^phox^ and p22^phox^ heterodimer) by patient neutrophils was confirmed by 7D5 mAb staining as measured by flow cytometry (n = 1). Historical healthy control neutrophils (isolated from fresh blood) are also depicted (mean + SD, n = 23). **B,** H_2_O_2_ release in response to opsonized serum-treated zymosan (STZ) (1 mg/mL), PMA (100 ng/mL), and PAF/fMLF (1 μM/1 μM) was measured by Amplex Red assay (n = 1). Historical healthy control neutrophils (isolated from fresh blood) are also depicted (mean + SD, n = 86). **C,** Nitroblue tetrazolium (NBT) microscopic staining of white blood cells from the travel control and the patient, stimulated with PMA (100 ng/mL) (original magnification ×400). **D,** Chemotaxis upon stimulation with C5a (10 nM), IL-8 (10 nM), PAF (100 nM), and fMLF (30 nM) measured over 3-μm pore size filters (mean + SEM, n = 2 of the patient and travel control, n = 1 of control and patient neutrophils isolated from bone marrow). Historical healthy control neutrophils (isolated from fresh blood) are also depicted (mean + SD, n = 15-74). *C5a*, Component 5a; *fMLF*, *N*-formylmethionyl-leucyl-phenylalanine; *MFI*, mean fluorescence intensity; *PAF*, platelet-activating factor; *PMA*, phorbol 12-myristate 13-acetate; *PMN*, polymorphonuclear cells; *RFU*, relative fluorescence units.

In view of the clinical picture and abnormal neutrophil morphology, further characterization of the myeloid compartment was undertaken. Maturation markers including CD11b, the alpha subunit of the major beta-2 integrin on neutrophils, as well as CD16 and CD66b were reduced on patient neutrophils (Fig 1, *D*). The patient neutrophils appeared phenotypically immature, lacking multilobed nuclei (Fig 1, *E*). Rather, the nuclei of these neutrophilic cells resembled those of metamyelocyte- or band neutrophils. Expression of cytochrome b558 (that is, the membrane component of the nicotinamide adenine dinucleotide phosphate [NADPH]-oxidase system), was confirmed by flow cytometry as was preservation of the neutrophil oxidative burst in response to phorbol 12-myristate 13-acetate; a nitroblue tetrazolium test result was also positive (Fig 2, *A-C*). However, there was a reduced neutrophil oxidative burst response to platelet-activating factor and *N*-formylmethionyl-leucyl-phenylalanine, consistent with an immature neutrophil phenotype that lacks expression of formyl peptide receptor-1^5^ (Fig 2, *B*). Strikingly, patient white blood cells and bone marrow–derived neutrophils showed severely impaired chemotaxis to complement component 5a, *N*-formylmethionyl-leucyl-phenylalanine, IL-8, and platelet-activating factor (Fig 2, *D*; see movies available online at https://dx.doi.org/10.17632/b94vwmvkz2.1).

**Fig 3:**
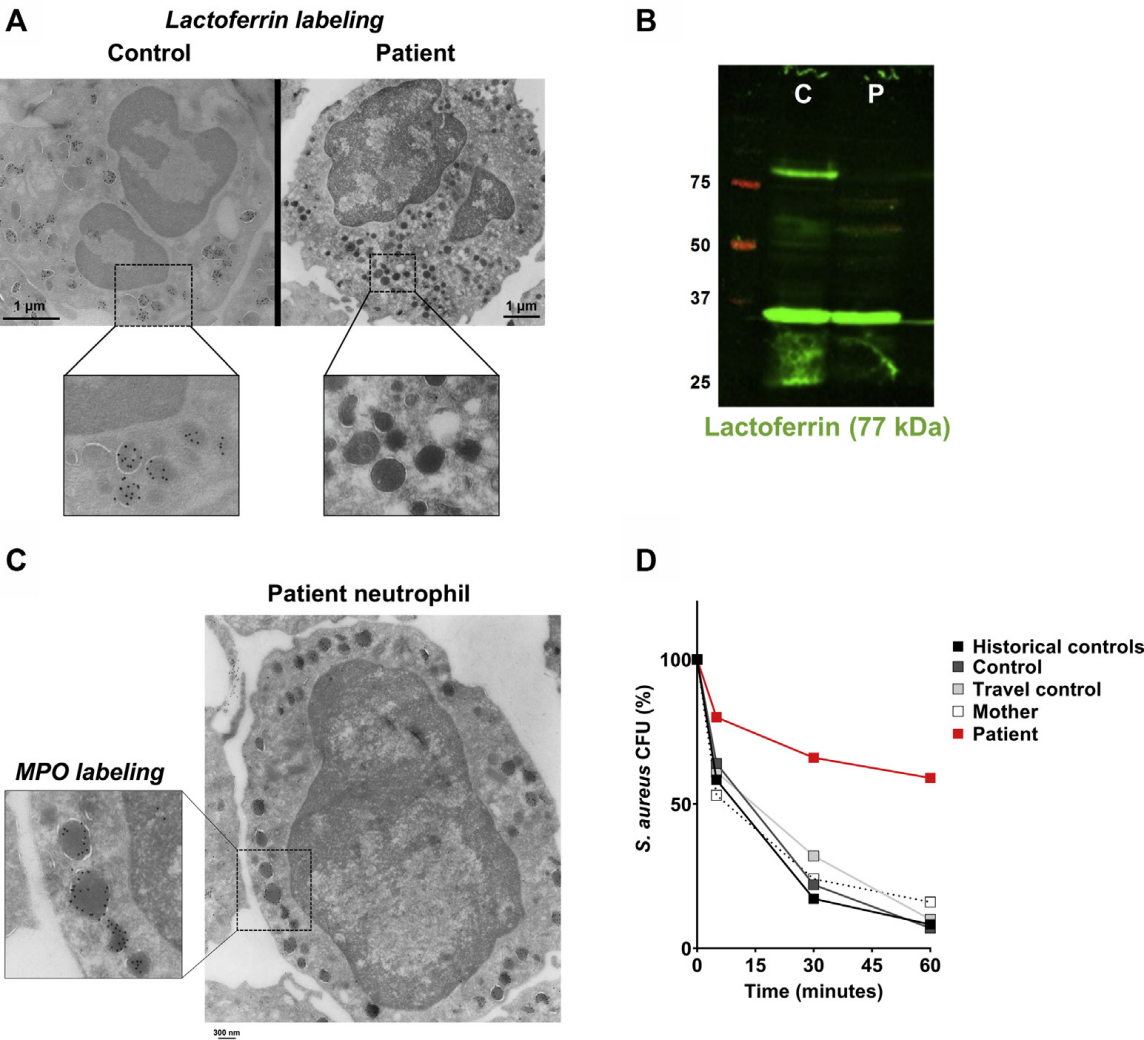
SMARCD2 deficiency results in SGD and defective bacterial killing by patient neutrophils. **A,** Representative immunoelectron microscopy image stained for lactoferrin (black dots). Images were acquired with a Tecnai12G2 electron microscope. Scale bar = 1 μm. **B,** Absence of lactoferrin (77 kDa) in patient white blood cells was observed by Western blotting. GAPDH (37 kDa) was used as loading control (C = control; P = patient). **C,** Representative immunoelectron microscopy image of a patient neutrophil stained for MPO (black dots). Image was acquired with a Tecnai12G2 electron microscope. Scale bar = 300 nm. **D,** Killing of opsonized *Staphylococcus aureus* was quantified by determining colony-forming-units at different time points (time = 0 minutes as 100%) (n = 1). Historical healthy control neutrophils (isolated from 1-day old blood) are also depicted (mean + SD, n = 30). *CFU*, Colony-forming-unit; *GAPDH*, glyceraldehyde 3-phosphate dehydrogenase; *MPO*, myeloperoxidase.

**Fig 4:**
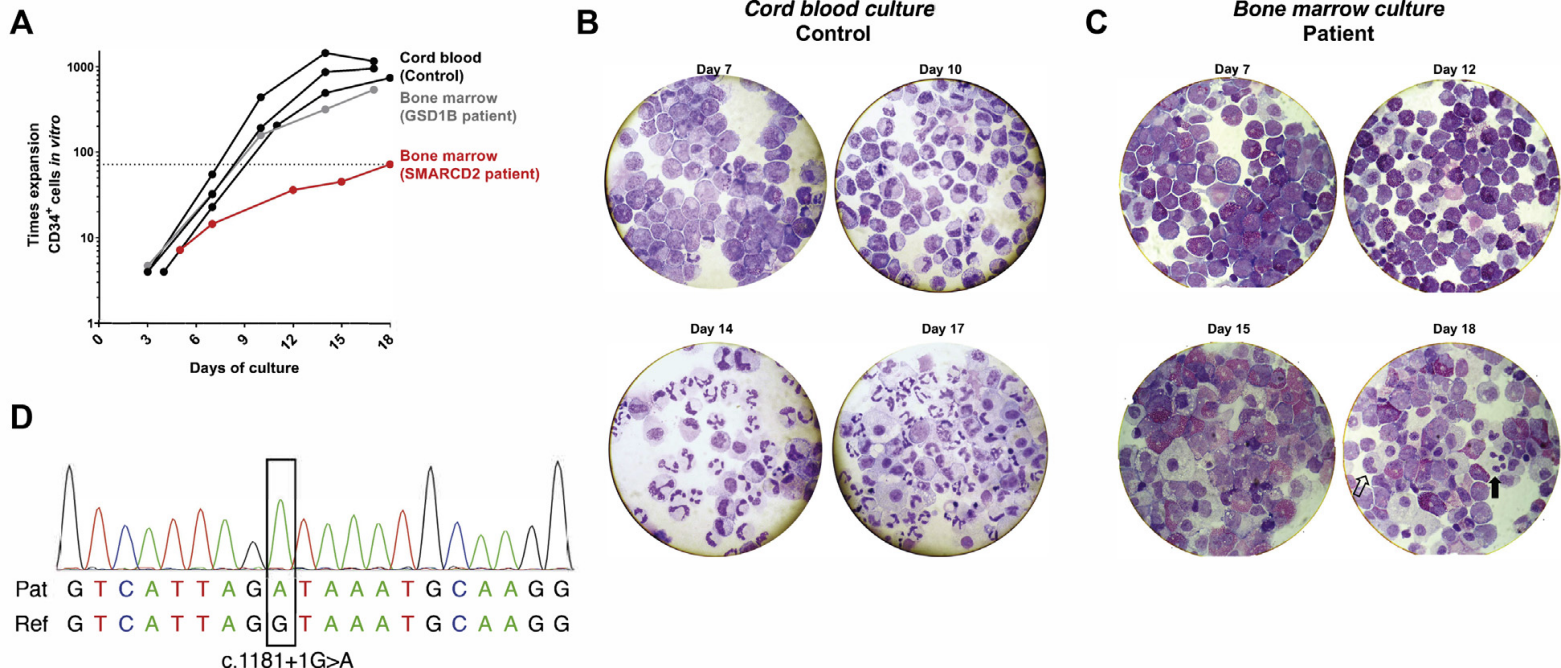
SMARCD2-deficient CD34^+^ cells fail to expand and differentiate toward the neutrophil lineage. **A**, *In vitro* CD34^+^ cell expansion toward the neutrophil lineage (time = days in culture). CD34^+^ cells were derived from control cord blood (n = 3) or bone marrow from the patient (n = 1) and an unrelated patient with glycogen storage disease type 1B (n = 1). **B** and **C**, Cytospins of *in vitro* CD34^+^ cell differentiation toward the neutrophil lineage on the indicated days. Cytospins shown for CD34^+^ cells derived from control cord blood (Fig 4, *B*) or bone marrow from the patient (Fig 4, *C*). Unfilled arrow indicates a band neutrophil, and black arrow indicates a segmented neutrophil (in the patient). **D,** Sanger sequencing confirming the patient’s homozygous variant at *SMARCD2* c.1181+1G>A.

Immunoelectron microscopy and immunoblotting revealed SGD based on absence of lactoferrin but normal myeloperoxidase staining in patient neutrophils (Fig 3, *A-C*). In keeping with a clinical phenotype of SGD,^6^ patient neutrophils showed defective *in vitro* killing of *S aureus* (Fig 3, *D*). Patient bone marrow–derived CD34^+^ cells showed markedly impaired *in vitro* expansion and differentiation toward the neutrophil lineage compared with CD34^+^ cells derived from control cord blood or bone marrow from an unrelated patient with glycogen storage disease type 1B (Fig 4, *A-C*). Taken together, these results suggested a severe abnormality of the myeloid compartment associated with loss of key neutrophil functions.

A clinical decision was made to proceed to matched sibling hematopoietic stem cell transplantation despite the lack of a molecular diagnosis. Aged 5.5 months, the patient was conditioned with treosulfan and fludarabine and received bone marrow containing 4.9 × 10^5^ CD34^+^ stem cells/kg, with ciclosporin and mycophenolate mofetil as graft versus host disease prophylaxis. The transplant itself was uneventful, but full chimerism was not achieved and slipped further despite an unconditioned top-up at 6 months (10 × 10^6^ CD34^+^ stem cells/kg). She was reconditioned at age 16 months with alemtuzumab, fludarabine, and busulfan (2.4 mg/kg with area under the curve 69.8 mg/L × h). She received a matched unrelated donor peripheral blood hematopoietic stem cell transplantation (19.8 × 10^6^ CD34^+^ stem cells/kg) and recovered without any major transplant-related complications at the time or since. She has learning difficulties and attends a school for children with special educational needs. Her weight is now on the 50th centile for age. Clinic reviews also note misaligned teeth and brittle nails.

In pursuit of a retrospective molecular diagnosis, whole-exome sequencing of patient genomic DNA samples taken pretransplant revealed homozygosity for a known pathogenic variant in *SMARCD2*, a chromatin-remodeling factor involved in the regulation of myelopoiesis. Mirroring our patient’s phenotype, the 4 patients recently described with autosomal-recessive deficiency of SMARCD2 also presented with delayed cord separation, recurrent infection, and SGD.^3^ The variant in our patient, which was confirmed by Sanger sequencing (Fig 4, *D*), disrupts an essential splice site of *SMARCD2* (c.1181+1G>A) and is shared by one of the kindreds described.^3^ Three different truncated *SMARCD2* mRNA transcripts were detected in patient-derived cells with the same variant, resulting from exon skipping and intron retention.^3^ As set out in Table I, multiple characteristics are shared between our patient and those previously described, including extrahematopoietic features such as learning difficulties, misaligned teeth, and brittle nails.^3^

**Table I:** Comparison of clinical and laboratory features in current and previously described patients with autosomal-recessive deficiency of SMARCD2

Clinical features in previously described patients	Clinical features in this patient
Clinical (4/4): • Delayed umbilical cord separation • Pneumonia • Recurrent septicemia • Intractable diarrhea • Failure to thrive	• Delayed umbilical cord separation • Poor wound healing • Sepsis • Diarrhea • Failure to thrive
Hematological findings (4/4): • SGD • Maturation arrest in bone marrow • Blast excess in bone marrow	• SGD
Extrahematopoietic: • Increased interdigital space D1-D2 (2/3) • Osteopenia (1/3) • Brittle, dysplastic, short nails (2/3) • Hirsutism (2/3) • Developmental delay, learning difficulties, dyspraxia (2/3) • Irregularly shaped misaligned teeth (partly conical) (2/2)	• Brittle nails • Learning difficulties • Misaligned teeth

In summary, we provide the second report of human deficiency of SMARCD2 leading to an immunodeficiency syndrome of delayed cord separation, infection, and SGD. We confirm the anticipated functional defects of chemotaxis and bacterial killing in primary SMARCD2-deficient neutrophils. Our results emphasize the role of SMARCD2 in neutrophil development and function and highlight the curative potential of hematopoietic stem cell transplantation for immunologic aspects of this condition.

**Key messages**

• SMARCD2 deficiency is characterized by SGD and defective neutrophil maturation as well as extrahematopoietic features, including learning difficulties, brittle nails, and misaligned teeth.
• Primary SMARCD2*-*deficient neutrophils are phenotypically immature and lack specific granules.

For detailed methods, please see the Methods section in this article’s Online Repository at www.jacionline.org.

## Methods

### Study subjects

Patients and their relatives provided written informed consent to participate in research protocols approved by the Newcastle and North Tyneside 1 Research Ethics Committee. Whole blood samples were obtained from these individuals, and genomic DNA was isolated using the DNeasy or QIAamp DNA mini kit (Qiagen, Manchester, UK). The study was approved by the local ethical committee of the Amsterdam University Medical Center and Sanquin Blood Supply (Amsterdam, The Netherlands).

### Isolation of primary cells

Whole leukocyte preparations were made by lysis of erythrocytes by ice-cold isotonic NH_4_Cl solution.^E1^ Cells were counted by a CASY Cell Counter, and cell concentrations were adjusted to 5 × 10^6^ neutrophils/mL. Cells were resuspended in HEPES medium (containing 132 mM NaCl, 20 mM HEPES, 6.0 mM KCl, 1.0 mM MgSO_4_, 1.0 mM CaCl_2_, 1.2 mM potassium phosphate, 5.5 mM glucose, and 0.5% (wt/vol) human serum albumin, pH 7.4).^E2^ Cytospins were prepared and stained with May-Grünwald/Giemsa staining to evaluate cell morphology and viability.

### Immunoelectron microscopy

For electron microscopy, whole leukocyte preparations were fixed in 2% (wt/vol) paraformaldehyde with 0.2% (wt/vol) glutaraldehyde and then processed for ultrathin cryosectioning. Cryosections (50 nm thick) were cut at −120°C with diamond knives (diatome) in a cryo-ultramicrotome (Leica, Vienna, Austria) and transferred onto carbon/formvar-coated copper grids. For immuno-labeling, the sections were incubated for 10 minutes with 0.15 mol glycine in PBS and for 10 minutes with 1% BSA in PBS to block free aldehyde groups and prevent nonspecific antibody binding, respectively. Sections were incubated with antibodies against human myeloperoxidase (rabbit polyclonal, A0398, Dako, Glostrup, Denmark) or against human lactoferrin (rabbit polyclonal, 02011092, Cappel Laboratories, Cochranville, Pa) followed by 10 nm protein A–conjugated colloidal gold (EMlab, University of Utrecht, The Netherlands) all in 1% BSA in PBS, and finally embedded in methylcellulose with 0.6% (wt/vol) uranyl acetate, and examined with a Tecnai12G2 electron microscope (Thermo-Fisher, Eindhoven, The Netherlands).

### Chemotaxis and TAXIScan analysis

White blood cell fractions were labeled with calcein-AM (1 μM; Molecular Probes, Eugene, Ore) for 30 minutes at 37°C, washed twice in PBS, and resuspended to a concentration of 2 × 10^6^ neutrophils/mL in HEPES medium. Chemotaxis in response to complement component C5a (10 nM; Sino Biological, Wayne, Pa), IL-8 (10 nM; PeproTech, London, UK), platelet-activating factor (100 nM; Sigma-Aldrich, St Louis, Mo), or formyl-Met-Leu-Phe (*N*-formylmethionyl-leucyl-phenylalanine; 30 nM; Sigma-Aldrich) was assessed with 3-μm pore-size Fluoroblock inserts (Corning, Inc, Corning, NY).

To visualize chemotaxis in response to component 5a (100 nM) and *N*-formylmethionyl-leucyl-phenylalanine (100 nM), TAXIScan analysis was performed with an EZ-TAXI scan (ECI, Inc, Tokyo, Japan) as previously described.^E3^

### NADPH-oxidase activity

The production of reactive oxygen species was assessed by measurement of the release of hydrogen peroxide with an Amplex Red assay (Molecular Probes) as described previously.^E3^ Cells were stimulated with serum-treated zymosan (STZ, 1 mg/mL; Santa Cruz, Dallas, Tex), phorbol 12-myristate 13-acetate (100 ng/mL; Sigma-Aldrich), or platelet-activating factor (1 μM) in combination with *N*-formylmethionyl-leucyl-phenylalanine (1 μM).

Data are expressed as the maximal slope in relative fluorescence units/minute. Nitro-blue tetrazolium slide reaction to evaluate superoxide production per individual cell was performed as described previously.^E4^

### *S aureus* killing

To assess bactericidal activity, neutrophils were incubated with opsonized *S aureus* and bacterial colony-forming units were determined as an indicator for bacterial survival as described previously.^E5^ The killing capacity is determined as a percentage, in which *t* = 0 is defined as 100%.

### Flow cytometry

Flow cytometry was performed to assess the surface expression of various neutrophil surface markers. Fluorescein isothiocyanate (FITC)-labeled mAbs and isotype controls were used according to the instructions of the manufacturer. Antibodies were: CD18-FITC (mouse IgG_1_ clone MEM48; Diaclone, Besançon cedex, France), CD11b-FITC (mouse IgM clone CLB-mon-gran/1, B2; Sanquin Reagents, Amsterdam, The Netherlands), CD16-FITC (mouse IgG_1_ clone 3G8; BD Biosciences, Franklin Lakes, NJ), CD66b-FITC (mouse IgG_1_ clone CLB-B13.9; Sanquin Reagents), and 7D5-FITC (mouse IgG_1_ clone 7D5; MBL Co, Nagoya, Japan). Samples were analyzed on a FACSCanto-II flow cytometer using FACSDiva software (BD Biosciences). Neutrophils were gated on the basis of their forward and side scatter. Data were analyzed with FlowJo (version 10.6.1) or FACSDiva software (both BD Biosciences).

### SDS-PAGE and Western Blot analysis

Samples were separated by SDS polyacrylamide gel electrophoresis and transferred onto a nitrocellulose membrane. Individual proteins were detected with antibodies against human lactoferrin (mouse monoclonal, clone 2B8; Abcam, Cambridge, UK) and against human glyceraldehyde-3-phosphate dehydrogenase (rabbit polyclonal; Merck Millipore, Darmstadt, Germany). Secondary antibodies were goat antimouse-IgG IRDye 800CW or goat antirabbit-IgG IRDye 800CW (LI-COR Biosciences, Lincoln, Neb). Quantification of bound antibodies was performed on an Odyssey Infrared Imaging system (LI-COR Biosciences).

### CD34-positive hematopoietic stem cell culture

CD34-positive hematopoietic stem cells were isolated from bone marrow or cord blood using CD34-MicroBeads (Miltenyi Biotec, Cologne, Germany). These cells were cultured toward the neutrophil lineage as is described in more detail by Aarts et al (manuscript in preparation, 2020). In short, CD34^+^ hematopoietic stem cells were cultured in Stemline II medium (Sigma-Aldrich) with 1% Pen/strep and a mix of cytokines and growth factors for 17 or 18 days. The cells were replated and received fresh media every other 3 days.

### Statistical analysis

Experimental data were plotted and analyzed by GraphPad Prism V8.02 (GraphPad Software, San Diego, Calif). Results are shown as mean ± range. Patient samples were analyzed once or twice (at different occasions).

### Video legend

Defective chemotaxis of patient cells assessed with TaxiScan. Travel control and patient cells stimulated with C5a (100 nM; Video 1 = travel control and Video 2 = patient) or fMLF (100 nM; Video 3 = travel control and Video 4 = patient). TAXIScan analysis was performed with an EZ-TAXI scan. Files can be viewed online in the Mendeley Data repository at https://dx.doi.org/10.17632/b94vwmvkz2.1. *C5a*, Component 5a; *fMLF*, *N*-formylmethionyl-leucyl-phenylalanine.
